# Towards the identification of essential genes using targeted genome sequencing and comparative analysis

**DOI:** 10.1186/1471-2164-7-265

**Published:** 2006-10-19

**Authors:** Adam M Gustafson, Evan S Snitkin, Stephen CJ Parker, Charles DeLisi, Simon Kasif

**Affiliations:** 1 Bioinformatics Graduate Program, Boston University, Boston, MA 02215 USA; 2 Department of Biomedical Engineering, Boston University, MA 02215 USA; 3 Children's Hospital Informatics Program of the Harvard MIT Division in Health Sciences and Technology, Boston, MA, USA

## Abstract

**Background:**

The identification of genes essential for survival is of theoretical importance in the understanding of the minimal requirements for cellular life, and of practical importance in the identification of potential drug targets in novel pathogens. With the great time and expense required for experimental studies aimed at constructing a catalog of essential genes in a given organism, a computational approach which could identify essential genes with high accuracy would be of great value.

**Results:**

We gathered numerous features which could be generated automatically from genome sequence data and assessed their relationship to essentiality, and subsequently utilized machine learning to construct an integrated classifier of essential genes in both *S. cerevisiae *and *E. coli*. When looking at single features, phyletic retention, a measure of the number of organisms an ortholog is present in, was the most predictive of essentiality. Furthermore, during construction of our phyletic retention feature we for the first time explored the evolutionary relationship among the set of organisms in which the presence of a gene is most predictive of essentiality. We found that in both *E. coli *and *S. cerevisiae *the optimal sets always contain host-associated organisms with small genomes which are closely related to the reference. Using five optimally selected organisms, we were able to improve predictive accuracy as compared to using all available sequenced organisms. We hypothesize the predictive power of these genomes is a consequence of the process of reductive evolution, by which many parasites and symbionts evolved their gene content. In addition, essentiality is measured in rich media, a condition which resembles the environments of these organisms in their hosts where many nutrients are provided. Finally, we demonstrate that integration of our most highly predictive features using a probabilistic classifier resulted in accuracies surpassing any individual feature.

**Conclusion:**

Using features obtainable directly from sequence data, we were able to construct a classifier which can predict essential genes with high accuracy. Furthermore, our analysis of the set of genomes in which the presence of a gene is most predictive of essentiality may suggest ways in which targeted sequencing can be used in the identification of essential genes. In summary, the methods presented here can aid in the reduction of time and money invested in essential gene identification by targeting those genes for experimentation which are predicted as being essential with a high probability.

## Background

A fundamental step in understanding how cells function is the comprehension of the minimal gene set required to sustain life. Before the core requirements for cellular life can be understood, it is necessary to identify the components of this set in diverse organisms. To date, prediction and discovery of essential genes has been supported by a significant amount of experimental work. Procedures such as single gene knockouts [1], RNA interference [2], and conditional knockouts [3] have been used as discovery mechanisms, but each of these techniques require a large investment of time and skill to perform. With the increase in availability of gene knockout data, many studies have been undertaken in an attempt to decipher the characteristics of essential genes. Through the analysis of essential genes in numerous organisms, fundamental evolutionary mechanisms and genomic fingerprints may be uncovered which will aid in essential gene identification in organisms lacking experimental validation.

Several studies have taken advantage of the abundance of experimental data available for model organisms in order to understand the properties of essential genes. For example, several groups have suggested that there is a relationship between degree in protein-protein interaction networks and essentiality [4,5]. The implication is that the hubs of the networks are of increased importance because of their abundance of interaction partners. Other studies have revealed relationships between essentiality and the number of transcription factor binding sites upstream of a gene [6]. It was demonstrated that those genes with more complex regulation are enriched in dispensable genes. High accuracy predictions of essential genes have also been made using flux balance analysis [7]. This method has the advantage of generating hypotheses regarding which genes are likely to be essential under a wide variety of hypothetical conditions. There is little doubt that with the plethora of experimental data being generated, additional properties of essential genes will be documented in the coming years.

While genome-wide experimental data is abundant in model organisms such as *S. cerevisiae *and *E. coli*, information is often limited for newly sequenced organisms, which precludes the use of such data for identification of genes essential for survival. The ability to identify essential genes in the absence of experimental data is of added importance, because it allows for a system to rationally select possible drug targets for newly sequenced pathogens. Fortunately, in addition to the relationships between essentiality and various experimental measures, there has been a good deal of research aimed at understanding the genomic features of essential genes. Metrics such as codon bias, number of paralogs, and phyletic retention have all been shown to be distinguishing of essential genes [8-10]. As essential genes are under a unique evolutionary pressure, it is likely that they share many other characteristics which may be gleaned from genome sequence data.

With both the practical and theoretical importance of the identification of essential genes in mind, we set out to construct an effective classifier of essential genes which exploited various genomic descriptors that could be generated directly form sequence data. Previous works aimed at understanding the properties of human disease genes have taken a similar approach [11,12]. Interestingly, many of the predictive descriptors of human disease genes identified by Kondrashov et al. [12] were identified in our study as being predictive of microbial essential genes.

As an initial step in the construction of our classifier, we explored various experimental and genomic metrics to assess how they relate to essentiality in both *E. coli *and *S. cerevisiae*. A metric which has been shown to be highly predictive of essentiality in previous studies was the retention of genes across different phyla. In order to extract the most from this metric we identified subsets of organisms achieving the highest accuracy in prediction of essential genes. The most predictive sets contained host-associated organisms with small genomes which are closely related to the query organism. This result directly suggests how targeted sequencing of genomes can be used in the prediction of essential genes. With the rapidly decreasing investments of both money and time required for the sequencing of a microbial genome, this approach of identifying essential genes through targeted sequencing may itself become a viable alternative in the identification of essential genes.

In addition to phyletic retention, we also assessed the relative performance of other previously reported indicators of essentiality such as protein interaction degree, protein size and codon bias. In *S. cerevisiae*, protein interaction degree was found to be highly predictive of essentiality, while protein size and codon bias were predictive in both organisms. Additionally, in *S. cerevisiae *we observed that high counts of certain individual charged amino acids were more predictive than size alone, implying that high counts of these amino acids distinguish proteins in a way not completely captured by their size.

Interestingly, although the aforementioned metrics were predictive of essentiality in both organisms, their relative importance and the sets of genes identified varied. The most striking difference is the relationship between protein size and essentiality. In *E. coli*, small proteins are enriched in essential genes, while in *S. cerevisiae *essential genes are underrepresented among the smallest proteins. We hypothesize that this may be indicative of a pressure in *E. coli *to maintain small proteins in the absence of other functional constraints, as has been previously suggested [13].

After identifying the genomic features most predictive of essentiality in *E. coli *and *S. cerevisiae*, we quantified the predictive limits of our assembled genomic characteristics by integrating them using a probabilistic classifier in conjunction with a feature selection criterion that is novel to bioinformatic applications. Using only easily obtainable genomic features from *S. cerevisiae *and *E. coli*, we show that our ability to predict essential genes is competitive with classifiers which included experimental features. The fact that we were able to construct our classifier using only descriptors generated from sequence data will allow broad application of this technique to other organisms, with only gene annotation being required. This ability has the potential to impact both the understanding of essential genes in different organisms, as well as the search for drug targets in poorly understood pathogens.

## Results and Discussion

### Experimental definition of essentiality

During our analysis of the relationship between various genomic features and essentiality, it is important to put our results in the context of our definition of essentiality. The definition used in most experiments is based on the growth, or lack thereof, of mutants under rich media conditions. Clearly, these conditions are not representative of the wild type environments which most of these organisms inhabited as they evolved their gene content. Studying metabolic pathways in *S. cerevisiae *using flux balance analysis indicates that as many as two-thirds of metabolic enzymes may be essential under some condition, while experiments in the presence of rich media result in roughly 20% of genes being labeled as essential [7]. Furthermore, analysis of the set of genes which have been labeled as dispensable in knockout experiments, which are also ubiquitously present throughout different phyla, revealed an overrepresentation of biosynthetic pathways [8]. It is likely that these pathways are essential under wild type conditions, as suggested by their retention throughout evolution, but with a surplus of nutrients provided they are identified as dispensable. Undoubtedly, there is room for debate as to a meaningful definition of essentiality, but it seems that, in general, most genes which are required under rich media conditions will be vital under most other conditions. Therefore, we feel that despite these inconsistencies, it is still valuable to understand the properties of this artificial set of essential genes, because although the comprehensiveness of the set can be questioned, its accuracy should only be limited by experimental bias.

### Selection of organisms for phyletic retention measure

The plummeting cost of genome sequencing is making comparative genomics an attractive technique, and creative bioinoformatic methods which take advantage of targeted sequencing are becoming more prevalent [14-16]. In this vein, we set out to understand the evolutionary properties of the sets of organisms in which the presence of an ortholog would be most indicative of the essentiality of a gene, with the hopes that by sequencing the appropriate genomes, high accuracy predictions of essential genes can be made. Retention of a gene over long evolutionary periods in a form that allows recognition using sequence similarity based techniques suggests that it is performing a critical function [17]. In previous studies, sets of organisms varying from a few distantly related organisms to several closely related organisms have been utilized in the examination of the relationship between the retention of genes and their essentiality [8,9]. To our knowledge, a systematic analysis with the aim of understanding the nature of a set of organisms in which presence of a gene is most predictive of essentiality has not been performed.

**Figure 1:**
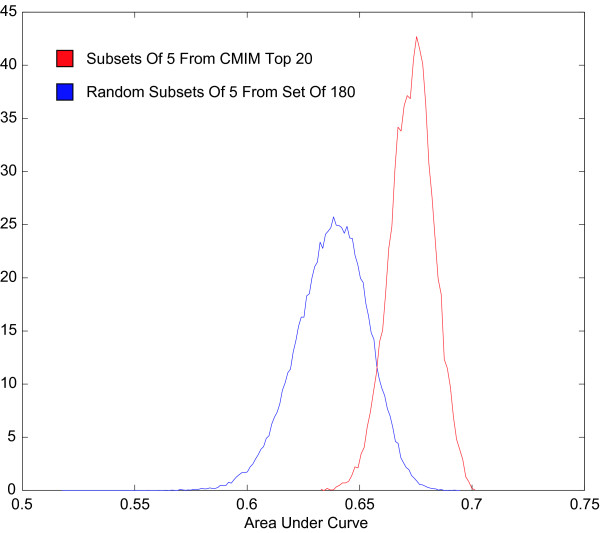
**Distribution of ROC areas under curves for *E. coli *essential gene prediction using CMIM selected subsets**. Probability density functions of the areas under curves from ROC analysis of 100,000 randomly selected subsets of five organisms selected from a total of 180 sequenced prokaryotes (blue) and all subsets of 5 organisms selected from a set of 20 which were in turn selected based on the conditional mutual information selection procedure described in the text (red). Sensitivity and specificity were computed based on the numbers of true positives (essential genes) and false positives (non-essential genes) identified as the number of organisms in which an ortholog of a given *E. coli *gene is present in decreases.

**Figure 2:**
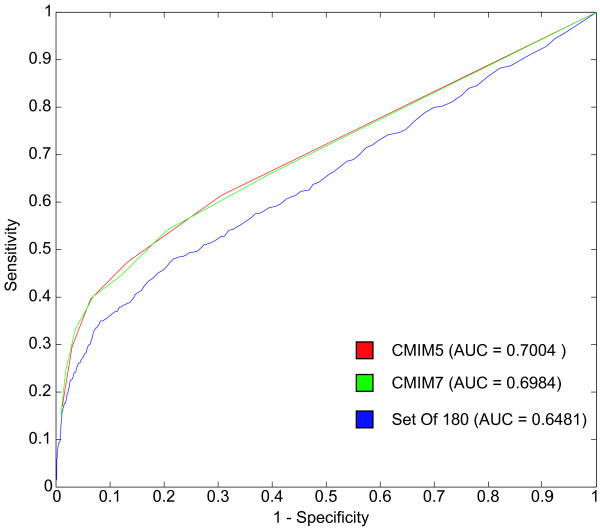
**ROC curves for different sets of organisms for *E. coli *essential gene prediction**. ROC curves were made based on the same methodology as those used in Figure 1. In red is the optimal set of 5 organisms selected from the CMIM set of 20, in green is the optimal set of 7 selected from the CMIM set of 20, and in blue is the set of 180 prokaryotes used in this study. The area under each curve (AUC) is indicated on the graph.

In order to identify the set of organisms in which the retention of a gene is most predictive of essentiality, we first selected a small group of organisms likely to be predictive for the *E. coli *essential list, and then computed the accuracy for all subsets of this reduced set. This strategy was employed because the large number of sequenced organisms prevented us from examining all possible subsets. In order to assemble our reduced set we ranked organisms based on the conditional mutual information maximization criteria (CMIM), as described by Fleuret [18] and detailed in the Methods section. Briefly, CMIM selection is an iterative feature selection procedure in which those features are selected which have the highest mutual information with the class variable, conditioned on those features which have already been selected. This procedure was implemented such that each organism is a feature, and the feature vector stores the occurrence of orthologs of *E. coli *genes. When defining the class variable as the essentiality of the corresponding genes, CMIM selection returns organisms that are highly predictive of essentiality as a set. Using this method we selected 20 organisms from a set of 180 sequenced prokaryotes and looked at the predictive accuracy for all possible subsets of various sizes. The utility of this approach was validated by comparing the distribution of Receiver Operating Characteristic (ROC) scores of our intelligently selected subsets to random sets of organisms (Figure 1), as well as to sets of organisms selected using maximal mutual information (data not shown). We also compared the performance of our optimal subset of five organisms with larger sets, and as can be seen in Figure 2 and Table 1, little is lost despite the small number of organisms used. Thus, by using only five optimally selected organisms we were able to achieve better performance than when using all 180 sequenced prokaryotes [see Additional file 5 for the organisms used in this analysis].

**Figure 3:**
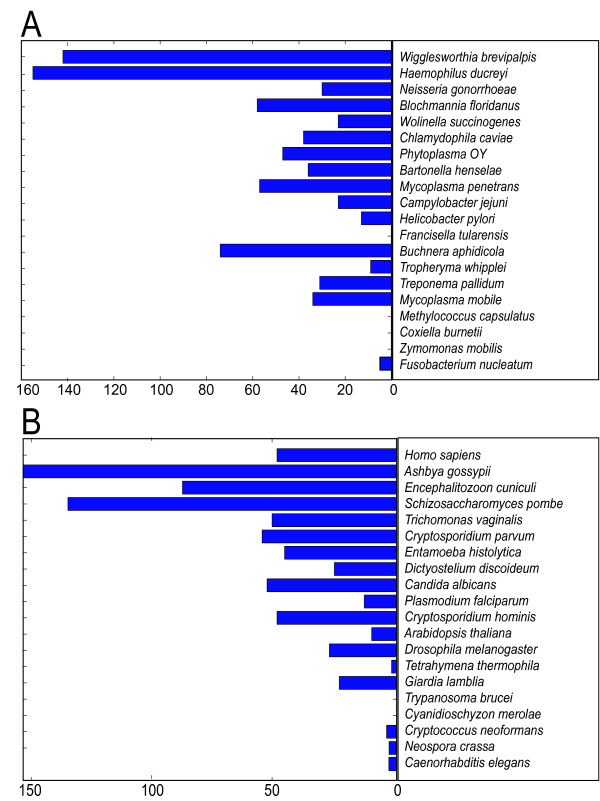
**Distribution of organism occurrence in the top performing organism sets**. Histograms were made which display the frequency of each of the CMIM selected top 20 organisms among the top 1% of organism sets of 5 in (a) *E. coli *and (b) *S. cerevisiae*. Note that organisms are displayed on the histograms from left to right based on their order of selection in the CMIM selection procedure. It can be seen in both graphs that there is a trend in which the organisms selected earlier are more often present in the best performing sets, which further validates the use of this selection method.

With evidence that our organism sets were among the best possible, we next examined the distribution of organisms among the most predictive sets. To do this we identified those sets of five organisms which achieved a ROC score in the top 1%, when compared to all subsets of five selected from our reduced set of 20. As can be seen in Figure 3A, among the organisms which are included most frequently in the best performing sets of five are *Buchnera aphidicola *and *Wigglesworthia glossinidia*. Both of these organisms are host-associated organisms belonging to the Gamma proteobacteria, to which *E. coli *also belongs. The explanation for the presence of orthologs in these organisms being the most predictive of essentiality in *E. coli *is clear when considering their evolutionary history and lifestyle. First, these organisms share a relatively recent common ancestor with *E. coli*, and during their adjustment to a host associated lifestyle they underwent massive gene loss, with minimal genetic novelty [19,20]. In the presence of this great selective pressure to minimize genome size, the majority of those genes retained were absolutely essential. One reason why several organisms perform better than one is because the gene set of any given reduced genome is partly dependant on the sequence of loss. In other words, the presence of alternative pathways allows for several viable minimal sets to arise during the process of reductive evolution, making an intersection of the gene sets of several organisms more informative than one [21]. The second property of these organisms which accounts for their superiority in prediction of essentiality relates to our working definition of essentiality being survival under rich media conditions. Although rich media conditions can be argued to be unrepresentative of wild type conditions for most organisms, these conditions are a fairly accurate representation of wild type conditions for organisms which resides inside a host, utilizing the available nutrients. Again, having several organisms is preferable, as the exact set of nutrients provided by the host will vary, and an intersection of gene sets best captures a generic host-associated lifestyle [20].

Performing similar analysis in *S. cerevisiae *using a set of 26 sequenced eukaryotes returned *Schizosaccharomyces pombe, Encephalitozoon cuniculi, Eremothecium gossypii *as the three most abundant organisms in the most highly predictive sets of five (Figure 3B). Both *E. cuniculi *and *E. gossypii *lead host-associated lifestyles, corroborating our interpretation of the optimal organism sets in *E. coli*. Furthermore, *E. gossypii *is among the smallest known eukaryotic genomes, with a 9.2 Mb nuclear genome [22].

### Performance of individual features in yeast

**Figure 4:**
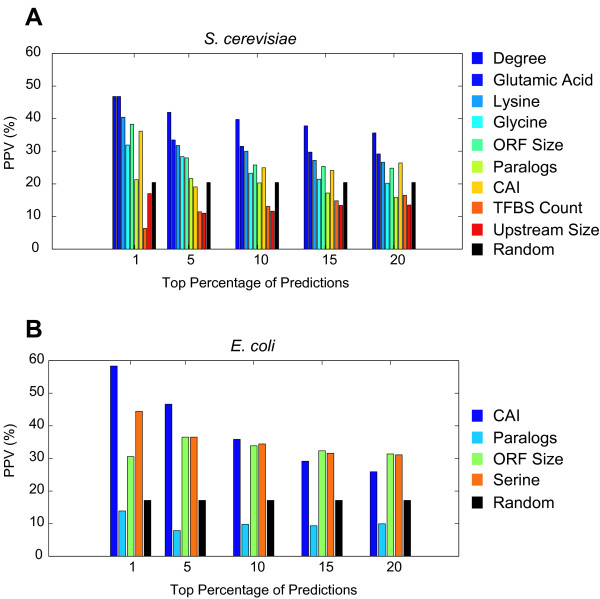
**Comparison of single feature classification accuracies of essential genes**. Displays the accuracy of single features in yeast (A) and *E. coli *(B) at classifying essential genes. Predictions are shown for cutoffs of the top 1, 5, 10, 15 and 20% (x-axis), with accuracy, as measured by PPV, shown on the y-axis. For predicting essentiality, all single features were ordered in descending order, with the exception of ORF size and Serine in the *E. coli *figure (B).

We extended our analysis of essential genes by gathering various data sets that are representative of different aspects of yeast biology so that we could quantify their abilities to distinguish essential from non-essential genes. Because we value the ability to predict essentiality in less studied organisms, our data sets were focused on features that are easily obtainable; that is, features that can be generated without the need for extensive laboratory work. These features fall into two general categories: (1) genomic features, which are based solely sequence data, and contain features such as open reading frame (ORF) size, upstream size, and phyletic retention; (2) protein features, which are based solely on protein characteristics and contain data sets like amino acid composition, codon bias, and hydrophobicity. As a reference for comparing the performance of our genomic and protein features, we included data sets derived from lab-intensive experiments such as protein-protein interaction and cellular localization data. The positive predictive value (PPV) of selected single features at varying coverage is displayed in Figure 4A. A complete list of the features used, as well as our rationale for including them, is detailed in Methods [see Additional file 3 for actual feature matrix used].

### Genomic features

As expected, the best performing genomic feature was the phyletic retention measure, whose construction was described above. For clarity, the term 'phyletic retention' was used to describe the presence of an ortholog in other organisms, in place of the term 'conservation,' in order to prevent confusion with measures of substitution rate. A second feature from our genomic set which was predictive of essentiality was the total upstream size of a gene. Genes with the largest upstream sizes are markedly enriched in dispensable genes. This result may be explained when considering the recent results by Yu *et al*. showing that genes with complex regulation are enriched in dispensable genes, in conjunction with the possibility that genes with more complex regulation may have larger upstream regions in order to accommodate an increased number of cis elements [8]. This connection has previously been shown to be valid in *Caenorhabditis elegans *and *Drosophila melanogaster *[23]. Our results suggest that a relationship exists between regulatory complexity and intergenic distance in *S. cerevisiae *and that this relationship accounts for the association between intergenic distance and essentiality. Given that the number of transcription factor binding sites present in the promoter of a gene is determined through arduous experimental procedures, it is beneficial to be able to use upstream size as a proxy for regulatory complexity.

### Protein features

Examining single features from the protein subset revealed several as being highly predictive of essentiality. In addition to previously discussed descriptors such as codon bias and protein size, we also identified enrichment in essential genes among proteins with an abundance of certain amino acids. Specifically, proteins with the highest counts of aspartate, glutamate, and lysine are enriched in essential genes with PPVs of 29.6%, 31.5% and 30.0% respectively in the top 10% of predictions. This trend is partially explained by observing that large proteins in general are enriched in essential genes, with a PPV of 25.8% among the largest 10% of proteins. Although this relationship in part explains high amino acid counts being predictive of essentiality, it fails to fully clarify why specific amino acids are more predictive than others. In order to gain insight into this phenomenon, we looked for enrichment in GO molecular function categories for proteins with high counts of charged amino acids that were not present among the largest 10% of proteins. Although no individual function attained a significant p-value, the functions present almost all involved either catalytic activity or an interaction with nucleic acids. Charged amino acids are often present in the active sites of enzymes, where they participate in catalytic mechanisms. Additionally, charged amino acids make associations with charged substrates more favorable due to electrostatic interactions. Based on these observations, we hypothesize that the enrichment in essential genes among those proteins with high counts of charged amino acids is in part because of the functional capabilities of these amino acids.

### Experimental features

In addition to examining the predictive power of features from our genomic and protein set, we also measured the prediction accuracy of some experimentally derived features previously reported to be indicative of essentiality. We observed that genes with a high degree in a protein interaction network are more likely to be essential, which is in agreement with previous work [5,24,25]. Among those proteins in the top 5% for degree, 42% are essential, a considerable enrichment in essential genes when compared to the ~17% expected by chance alone. It should be noted that it has been stated in the literature that the relationship between degree and essentiality is at least partly due to biases in the data [26]. Specifically, Coulumb *et al*. state that the protein interaction dataset from the Database of Interacting Proteins (DIP), which we used in this analysis, is biased towards essential genes due to the accumulation of interactions from small scale experiments which are partial towards essential genes. The authors go on to state that this partiality accounts for a significant component of the relationship between degree and essentiality. This contention was then substantiated by showing the disappearance of the relationship between essentiality and connectivity when using unbiased whole-genome yeast two-hybrid experiments. Others have recently stated that the lack of reliability and completeness of yeast two-hybrid data are responsible for the absence of a relationship between connectivity and essentiality [27].

### Performance of individual features in *E. coli*

Despite a large evolutionary distance and fundamental biological differences, the characteristics of essential genes in *S. cerevisiae *and *E. coli *are largely similar. As seen in Figure 4B, the strongest predictor of essentiality in *E. coli *other than phyletic retention, is CAI. Number of paralogs and protein size were also predictive of essentiality in both organisms. Features for all *E. coli *genes used in this study are available in Additional files [see Additional file 4].

### Differences in feature performance between *S. cerevisiae *and *E. coli*

Although most of the features performed comparably in both organisms, there were some noticeable differences. For example, although features such as protein size and codon bias were predictive of essentiality, their accuracy as well as the sets of proteins which they identified varied between the two organisms.

**Figure 5:**
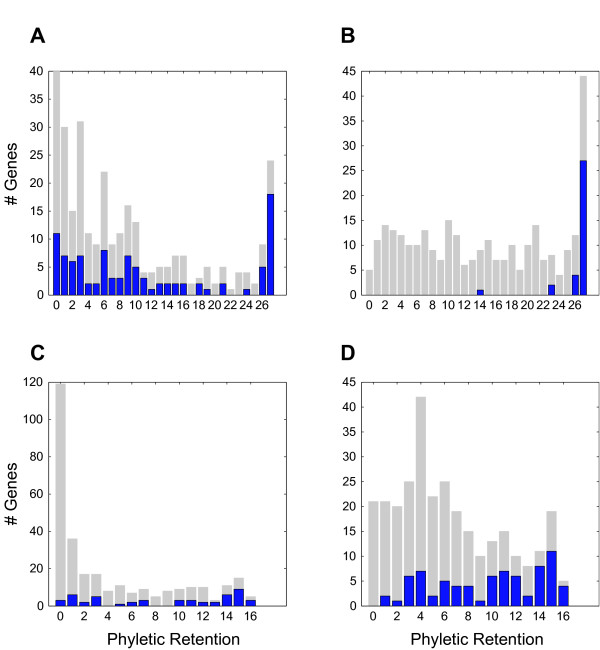
**Phyletic distribution of the smallest and largest essential genes in *S. cerevisiae *and *E. coli***. Displays the number of essential genes (blue) superimposed on the total number of genes (gray), binned by number of organisms in which the gene is present (phyletic retention) for (A) the 300 smallest proteins in *E. coli*, (B) the 300 largest proteins in *E. coli*, (C) the 300 smallest proteins in *S. cerevisiae *and (D) the 300 largest proteins in *S. cerevisiae*.

Protein size was predictive of essentiality in both organisms. Upon further analysis we determined that small proteins in *E. coli *are enriched in essential genes, while small proteins in *S. cerevisiae *are slightly enriched in dispensable genes. In order to gain insight into this discrepancy between the two organisms we examined the distribution of essentiality in small and large proteins in both organisms in the context of their phyletic retention. As seen in Figure 5, there is a marked difference in the distribution of large and small essential genes when dissected into bins based of their phyletic retention. In *E. coli*, virtually all of the largest proteins which are essential are ubiquitously present throughout the 27 sequenced non-parasitic Gamma proteobacteria we assembled (Figure 5B). We hypothesize that the enrichment of essential genes only among the most conserved large proteins may be indicative of a pressure to reduce the size of individual proteins. Larger proteins come at an increased cost to the cell in terms of both raw materials and energy expenditures during protein synthesis. This idea is consistent with findings from Lipman *et al*., where they observed increased conservation among the largest proteins in several genomes and attribute this to a pressure to maintain small proteins in the absence of other functional constraints [13]. The overall enrichment of essential genes among small proteins at all levels of conservation and the most conserved large proteins are both supportive of this hypothesis.

Figure 5C yields insight into the lack of essential genes among the smallest *S. cerevisiae *ORFs. The enrichment of dispensable genes amid small ORFs in *S. cerevisiae *seems to be a consequence of an abundance of small species specific genes. In both organisms species specific genes are enriched in dispensable genes, as would be expected based on the predictive power of phyletic retention in identifying essential genes. Therefore, an abundance of small species specific genes leads to the apparent trend of dispensability among the smallest *S. cerevisiae *genes. It should be noted that the organisms used in this phyletic conservation analysis were a set of 16 sequenced fungi and protists, so as to have a more diverse set of genomes than used in the optimal predictive set.

### Performance of integrated features in yeast

To determine the limits of our predictive abilities in the classification of essential genes when integrating multiple features, we utilized naïve Bayes classifiers. We assigned all of our features into three different overlapping sets, in order to assess the relative contributions of different subsets of features. The first set, which we will designate as SC_GenProt, is composed of all features which can be obtained directly from sequence data. Our second set, which is designated as SC_GenProt_No, is identical to SC_GenProt, but lacks the phyletic retention measure. We included this set in order to assess our ability to identify less conserved essential genes. Our third set, designated as SC_All, is composed of features that require extensive experimentation, in addition to all easily obtainable features, so that we could assess the impact of neglecting experimental data on our prediction accuracy. A benefit to using naïve Bayes for feature integration is that each classification is assigned a probability, making it natural to rank the predictions, which allows for direct comparison to results using individual features.

Feature selection was accomplished by ranking features using conditional mutual information maximization (CMIM), as described in Methods [see Additional file 1 for the actual ranking]. The phyletic retention feature achieved the highest mutual information with essentiality, which is consistent with our results on single feature performance. By using the 21 most informative features in SC_All, 11 in SC_GenProt and 13 in SC_GenProt_No, we were able to improve prediction accuracy over the inclusion of all features in each set [see Additional file 2].

**Figure 6:**
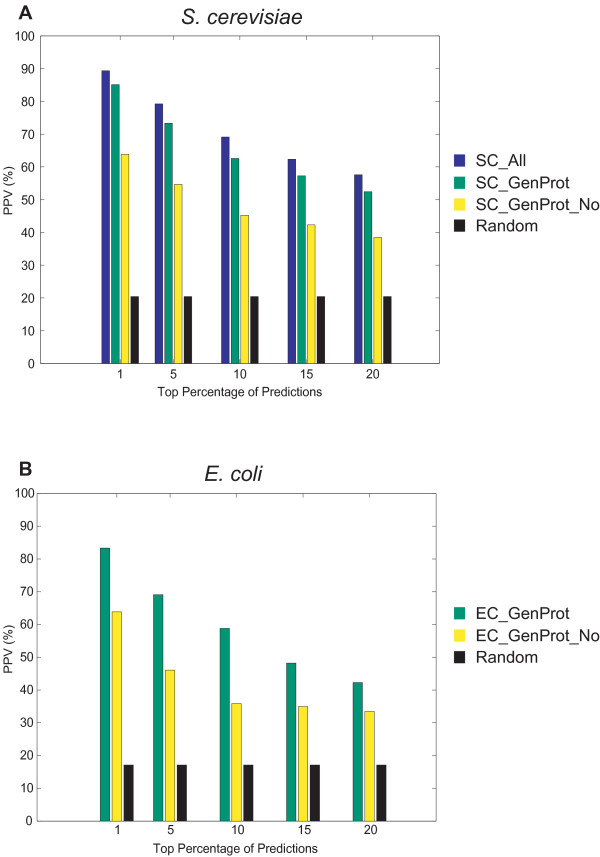
**Comparison of accuracies of naïve Bayes classifiers**. The predictive accuracies of integrating optimal feature sets using a naïve Bayes classifier are shown for yeast (A) and *E. coli *(B). Accuracy, as measured by PPV (y-axis) is shown for the top 1, 5, 10, 15 and 20% of predictions for the different feature sets.

Overall performance can be seen in Figure 6A, where the positive predictive value (PPV) in the top 1, 5, 10, 15 and 20% of predictions is shown in reference to selected single features. Unexpectedly, while SC_All outperforms SC_GenProt, it is only by a small amount (~5% difference at the top 15 and 20% of predictions, Figure 5A), which indicates that we are losing little by ignoring features derived from experimental data. As expected, SC_GenProt_No was greatly outperformed by the other feature sets. This again is consistent with the results shown in our single feature analysis, where the phyletic retention measure was by far the most predictive feature.

As SC_GenProt_No is performing significantly worse than other feature sets, it is only of use if it is identifying especially interesting genes. To assess the ability of SC_GenProt_No to identify essential genes that are less conserved, we looked at the broader conservation pattern of yeast genes in a set of 16 fungi and protists. Based on this set of 16 organisms, there were 285 essential yeast genes that had orthologs in 5 organisms or less. In the top 15% of predictions made by SC_GenProt_No, 24.6% of the 285 less conserved essential genes were identified. In contrast, only 3.5% of the 285 less conserved essential genes were identified by SC_GenProt at the same cutoff. Thus, while SC_GenProt_No has the lowest accuracy of the integrated feature sets, it is useful because of its increased ability to predict less conserved essential genes.

### Performance of integrated features in *E. coli*

As in yeast, feature sets integrated with a naïve Bayes classifier were used to predict essentiality in *E. coli*. Two sets containing easily obtainable features were analyzed, EC_GenProt and EC_GenProt_No. No experimental feature set was used due to a lack of available genome wide analyses.

The phyletic retention measure was, as in yeast, found to be the most informative feature when ranking by conditional mutual information [Additional file 1]. However, where in yeast we obtained the best PPV when using 13 features, *E. coli *required only the top four: phyletic retention, serine, tryptophan and paralog count (9 features were found to optimally classify EC_GenProt_No). Figure 6B shows the performance of the integrated features in *E. coli*, where PPV is shown for the top 1, 5, 10, 15 and 20% of predictions.

## Conclusion

The identification of essential genes has largely been an experimental effort, achieved through whole-genome knockout techniques. While in some organisms such as *C. elegans*, it is possible to devise highly effective screens for essential genes using siRNAs [28], the cost and/or ineffectiveness of this technique in other organisms makes its broad application currently infeasible. In this paper we assessed the potential effectiveness of a methodology in which genes are first prioritized based on their likelihood of testing positively in a lethality screen and after which subsequent small scale knockout screens can be performed on the top predictions to obtain experimentally validated genes.

We investigated the efficacy of this strategy by using available knockout experiments to assess the predictive power of features that are easily obtainable from sequence data and then integrating them using machine learning methodologies. By integrating genomic and protein characteristics of varying predictive power using a probabilistic classifier with feature selection, we were able to achieve an overall predictive accuracy in both *S. cerevisiae *and *E. coli *that was superior to the performance of any individual feature. The use of several descriptors will make our classifier more robust than using individual features whose predictive power is likely to vary a great deal among different organisms. For example, codon bias is a strong predictor of essential genes in both organisms studied here, but a study of 80 bacterial genomes revealed that 30% have no codon bias [29]. Furthermore, we were able to classify essential genes with a reasonable accuracy even without the use of a gene conservation measure such as phyletic retention, providing the added benefit of identifying essential genes which may be organism specific. The ability to identify essential genes from sequence data alone has the potential to be of great practical importance in guiding the investigations of researchers searching for potential drug targets in newly sequenced pathogens.

In the process of constructing an integrated classifier, the relationship between various genomic characteristics and essentiality were explored. In both *E. coli *and *S. cerevisiae*, phyletic retention, protein size, and codon bias were identified as being among the single features most predictive of essentiality. Furthermore, we showed that the most predictive groups of organisms used in a phyletic retention measure contain host-associated organisms which are closely related to the reference organism. Despite the influence of our artificial definition of essentiality on the selection of our optimal genome sets, this result is still useful in suggesting how targeted sequencing can be used in the identification of essential genes in other organisms. In addition to phyletic retention and codon bias, which have been related to essentiality in previous studies, we identified a relationship between protein size and essentiality, which to our knowledge has not been explored before. Specifically, we observed that the nature of this relationship differed for *E. coli *and *S. cerevisiae*, with small protein size being indicative of essentiality in *E. coli *and the same being true of large proteins in *S. cerevisiae*. Moreover, among the largest *E. coli *proteins, only those which are the most conserved are essential. We hypothesize that these observations are both indicative of a pressure to maintain a small proteome in *E. coli*.

In summary we have made strides towards the prediction of essential genes based solely on sequence data on two fronts. First, we have gained insight into the properties of sets of organisms in which the presence of an ortholog is most predictive of essentiality. Second, we have assessed the predictive power of several sequence based features, and achieved superior prediction accuracy through integration with a probabilistic framework and intelligent feature selection.

## Methods

### Sets of essential genes

Essential gene definitions were taken from Giaever et al[1] and Gerdes et al[30] for *S. cerevisiae *and *E. coli *respectively. Additionally, for *E. coli *all ORFs were removed which were less than 80 amino acids. For *S. cerevisiae *all ORFs were removed whose FASTA headers contained the key words "transposable" or "mitochondrial". In total 4,728 yeast genes were used, 966 of which are essential. In *E. coli*, 3569 genes were used, of which 611 are essential.

### Selection of Features

*S. cerevisiae *and *E. coli *are both model organisms which have been very well studied over the years. We capitalized on this fact by assessing the relationship among a variety of gene properties and essentiality. Following is a list of predictors and our rational for including them. Note that those features used in just *S. cerevisiae *are marked with a star, and those used only in *E. coli are marked with two stars*.

### Experimental Characteristics

The following parameters require a large amount of experimental work to obtain. These features were only used in our classifiers integrating all features, and were excluded from those using only 'easily obtainable' features.

#### *Protein interaction network degree

Generated from the curated interactions accumulated in the Database of Interacting Proteins (DIP) [31]. The degree of a protein is computed by summing the number of unique interactions which it participates in. Degree, along with related metrics of network position, have been documented in the literature as being indicative of essentiality [5,24,25]. Protein interaction data was not included for *E. coli *due to low coverage.

#### *Protein Localization

It is known that proteins with GO transcriptional regulation annotations are enriched in essential genes [1]. Based on this result we tested whether or not nuclear localization, along with other protein localization categories, are useful in predicting essentiality. Localization information was obtained from a previous large-scale study [32].

#### *Recombination Rate

Clusters of essential genes are known to be in regions of the genome that are characterized by a lower recombination rate [33]. All per-gene recombination rates were acquired from Gerton *et al*. [34], and analyzed according to procedures used by Pal and Hurst [33].

### Genomic Characteristics

We consider the following parameters 'easily obtainable' in that they can be automatically generated from sequence data.

#### Gene size

There is a trend for proteins to become larger throughout evolution. We therefore expected that gene size may be indicative of essentiality, especially in *E. coli*, as ancestral genes are likely essential.

#### *Regulatory complexity

Different genes in *S. cerevisiae *exhibit wide variation in their regulatory complexity. It has recently been documented that there is a relationship between regulatory complexity and essentiality [6]. We measured regulatory complexity using the following parameters: upstream size, downstream size, upstream conservation and downstream conservation. All sizes were measured as the distance to the nearest gene. Conservation was measured as the number of bases among the (up to) 1000 bp upstream of the ORF start site and (up to) 300 bp downstream of the designated open reading frame, that overlap with elements identified as being conserved in a seven species comparison (downloaded from the UCSC Genome Browser; most conserved track).

#### Phyletic retention measure

Genes that are ubiquitously present across different taxa are more likely to be essential [1]. As detailed in the Results section, for yeast and *E. coli *separately, a set of five organisms were selected that optimally predicted essentiality. A count was made for each gene in the reference organism (yeast or *E. coli*) that represents the number of orthologs present in the five organisms. Bi-directional best BLAST hits were used to define an orthologous relationship (using an E-value cutoff of 0.1).

#### Number of paralagous genes

Genes that do not have duplicates are more likely to be essential [1]. The rational is that the duplicates may function in a backup capacity in the presence of a knockout mutation in the original gene. Paralogs were defined as those genes which were present in the same genome which had a BLASTP E-value less than 10^-20. ^In addition the ratio of the larger gene to the smaller could not exceed 1.33.

#### **Strand bias

Essential genes are more likely to be encoded on the leading strand of the circular chromosome [35].

### Protein Characteristics

These parameters fall in our 'easily obtainable' category as well because they require coding sequence only; no laboratory experiments are necessary. We represent protein characteristics in terms of the following metrics: amino acid composition, codon bias, codon adaptation index (CAI), frequency of optimal codons (FOP), isoelectric point (PI), hydropathicity score, and hydrophobicity score. For yeast all data was downloaded from the Saccharomyces Genome Database [36]. For *E. coli *these metrics were generated using the CodonW software package [37].

### Integration of features

Classification of essential genes was done using the Orange machine learning package's implementation of naïve Bayes. [38]. All features, with the exception of the optimized phyletic retention measure and the binary localization features, were discretized using Fayyad and Irani's entropy discretization method [39], as implemented by Orange.

After discretization, conditional mutual information maximization criteria (CMIM), as described by Fleuret [18], was used to rank the features. Briefly, CMIM is an iterative method where feature vectors are selected that have the highest mutual information with the class vector after conditioning on previously selected features vectors.

Let *Y *be our class vector, *X*_*n *_be a feature, *I(Y;X*_*n*_) be the mutual information between *Y *and *X*_*n*_, and *I(Y; X*_*n*_* | X*_*m*_) be the conditional mutual information between *Y *and *X*_*n *_conditioned on *X*_*m*_. CMIM was implemented as follows, with alternate implementations described by Fleuret [18]. A score table *s *is initialized with the values *I(Y ; X*_*n*_). The algorithm picks at each iteration the feature *Xm *with the highest score, and then refreshes every score *s *[*n*] by taking the minimum of *s *[*n*] and *I *(*Y *; *Xn *| *Xm*). The algorithm is run until all features have been selected.

For a given feature set, the optimal number of features to include in classification was determined empirically. Features were ranked by CMIM, and iteratively removed one at a time. At each interval the classification accuracy was measured. The cutoff for the optimal feature set was identified as that with the highest PPV for the top 5% of predictions, with the requirement that PPV for the top 1% must be higher [see Additional file 2].

To assign the probability of essentiality to all genes, the following procedure was used. Half of all essential and half of all non-essential genes were randomly chosen to be included in the training set. The classifier was then tested on the remaining genes, which assigns a probability of essentiality. Training/testing was bootstrapped 100 times, and for each gene the probability of essentiality was taken as an average of all the probabilities that were assigned it [see Additional file 6].

### GO and KEGG enrichment analysis

The GeneMerge software was used to calculate enrichment of KEGG annotations, using a background of all genes used in the given organism [40]. P-values given are Bonferroni corrected.

## Authors' contributions

AMG, SCJP, ESS and SK conceived the project and designed the study. AMG and ESS drafted the manuscript. SCJP and ESS performed analysis of single feature performance. AMG performed integrated classifier analysis. ESS performed analysis on identifying optimal organism sets. CD and SK directed the research, and provided feedback throughout the project. All authors read and approved the final manuscript.

## Supplementary Material

Additional File 5 **List of organisms used to calculate phyletic retention**. A list of organisms used in the calculation of phyletic retention is shown. KEGG three letter codes are used to represent the organisms, unless otherwise noted. Click here for file

Additional File 3 **Feature matrices for *S. cerevisiae***. A raw data feature matrix, as well as an entropy discretized feature matrix are included. Click here for file

Additional File 4 **Feature matrices for *E. coli***. A raw data feature matrix, as well as an entropy discretized feature matrix are included. Click here for file

Additional File 1 **CMIM feature ranking**. This excel file includes tables showing the CMIM feature ranking. Click here for file

Additional File 2 **Performance of naïve Bayes classifiers using subsets of features**. For each set of features analyzed in this paper (e.g. SC_GenProt, EC_GenProt, etc...), CMIM was calculated such that features were ranked in order of most informative to least. PPV for the top 1, 5, 10 and 15% of predictions are shown when naïve Bayes classifier is constructed when using the top N features. Click here for file

Additional File 6 **Naïve Bayes classification results**. For each of the feature sets used on *E. coli *and *S. cerevisiae*, the probability of a gene being essential, as reported by naïve Bayes, is provided. Click here for file

## Figures and Tables

**Table 1:** Influence of organism composition on phyletic retention performance

**Organisms Present**	**Cumulative PPV**	**Number Of Genes in Group**	**Number of Essential Genes**
**CMIM Selected Set Of 5**			
5	74.8%	139	104
4	66.8%	271	181
3	55.6%	435	242
2	42.0%	690	290
1	28.9%	1302	376
0	16.8%	3636	612
**CMIM Selected Set of 7**			
7	77.0%	122	94
6	73.3%	206	151
5	65.3%	311	203
4	54.1%	455	246
3	43.0%	633	272
2	34.8%	952	331
1	25.1%	1613	405
0	16.8%	3636	612
**Set of 27 Non-parasitic Gamma Proteobacteria**			
27	59.0%	305	180
24	46.6%	545	254
20	34.5%	861	297
16	27.3%	1247	341
12	23.9%	1697	405
8	20.3%	2235	453
4	18.3%	2845	521
**Set of 179 prokaryotes**			
179	75.0%	12	9
161	75.3%	77	58
143	64.8%	196	127
126	54.4%	294	160
108	48.4%	419	203
90	39.7%	572	227
72	33.0%	817	270
54	26.8%	1172	314
37	22.4%	1659	371
19	19.2%	2379	457
1	17.0%	3516	596
